# Zebrafish as an In Vivo Model to Assess Epigenetic Effects of Ionizing Radiation

**DOI:** 10.3390/ijms17122108

**Published:** 2016-12-15

**Authors:** Eva Yi Kong, Shuk Han Cheng, Kwan Ngok Yu

**Affiliations:** 1 Department of Physics and Materials Science, City University of Hong Kong, Hong Kong, China; yikong3-c@my.cityu.edu.hk; 2 Department of Biomedical Sciences, City University of Hong Kong, Hong Kong, China; 3 State Key Laboratory in Marine Pollution, City University of Hong Kong, Hong Kong, China

**Keywords:** zebrafish embryos, epigenetic effects, ionizing radiation

## Abstract

Exposure to ionizing radiations (IRs) is ubiquitous in our environment and can be categorized into “targeted” effects and “non-targeted” effects. In addition to inducing deoxyribonucleic acid (DNA) damage, IR exposure leads to epigenetic alterations that do not alter DNA sequence. Using an appropriate model to study the biological effects of radiation is crucial to better understand IR responses as well as to develop new strategies to alleviate exposure to IR. Zebrafish, *Danio rerio*, is a scientific model organism that has yielded scientific advances in several fields and recent studies show the usefulness of this vertebrate model in radiation biology. This review briefly describes both “targeted” and “non-targeted” effects, describes the findings in radiation biology using zebrafish as a model and highlights the potential of zebrafish to assess the epigenetic effects of IR, including DNA methylation, histone modifications and miRNA expression. Other in vivo models are included to compare observations made with zebrafish, or to illustrate the feasibility of in vivo models when the use of zebrafish was unavailable. Finally, tools to study epigenetic modifications in zebrafish, including changes in genome-wide DNA methylation, histone modifications and miRNA expression, are also described in this review.

## 1. Introduction

All living organisms including humans are continuously exposed to ionizing radiations (IRs) that can be categorized into terrestrial, cosmic and man-made radiations. IR is a significant genotoxic agent that can generate deleterious deoxyribonucleic acid (DNA) lesions leading to serious consequences such as cancer induction [1]. The biological effects of IR can be categorized into “targeted” and “non-targeted” effects, both effects being involved in radiation damage to cells. While the “targeted” effect occurs in the irradiated cells or organisms, a “non-targeted” effect refers to the propagation of effects from irradiated cells or organisms to non-irradiated or bystander cells or organisms. The IR-induced targeted and non-targeted effects will be briefly reviewed in Section 2.

In recent years, transgenerational effects of IR have attracted a lot of research interest. In relation, there have been extensive studies on IR-induced epigenetic effects which do not involve alterations in the DNA sequence. These effects modulate gene expression patterns without modifying the actual DNA sequence, and as such can lead to heritable changes in gene expression not encoded by the DNA sequence [2]. Epigenetic alterations have profound effects on a range of cellular expressed genes [3]. DNA methylation, histone modifications and microRNA profiles will be discussed in Section 3.1, Section 3.2 and Section 3.3.

The present paper reviews the potential of zebrafish, *Danio rerio*, as an in vivo model to assess epigenetic effects of IR. Zebrafish, *Danio rerio*, is a scientific model organism that has yielded scientific advances in several fields. The zebrafish genome has been assembled at high resolution [4,5]. Zebrafish share 70% of their genes with human beings, including conservation of most DNA repair-related genes [6]. The popularity of this model is also due to the transparency of early embryos, the rapid development and well-characterized developmental stages, as well as convenient husbandry. Section 4 will describe epigenetic effects of IR in zebrafish. The use of zebrafish as a model organism in various studies will be briefly outlined in Section 4.1 while the epigenetic effects in zebrafish will be reviewed in Section 4.2. DNA methylation, histone modifications and miRNA profiles in regulating IR effects in zebrafish will be discussed in Section 4.3, Section 4.4 and Section 4.5. Studying epigenetic effects of IR using zebrafish is still in its infancy, and therefore results have not yet been as plentiful as expected. Other in vivo models are included to compare observations made with zebrafish, or to illustrate the feasibility of in vivo models when the use of zebrafish was unavailable. Wherever necessary and appropriate, descriptions of in vitro studies will also be included. Interestingly, most applicable information was available in and before 2013. Finally, Section 5 will give a brief summary.

## 2. Ionizing Radiation, Targeted Effects and Non-Targeted Effects

Traditionally, detrimental effects of IRs were thought to occur only in the directly irradiated nuclear targets, i.e., the effects were restricted to “targeted” effects. DNA double-strand breaks (DSBs) induced by IRs are considered the most relevant lesion for mutations and carcinogenesis, and unrepaired or mis-repaired DSBs are a serious threat to genomic integrity [7,8]. Since the 1990s, “non-targeted” effects of IRs, i.e., the propagation of effects from irradiated cells or organisms to non-irradiated or bystander cells or organisms, were revealed and characterized. These included the bystander effect, adaptive response, rescue effect, genomic instability, changed profiles of gene expression and of non-coding RNAs. Radiation-induced bystander effects (RIBEs) refer to biological effects which occurred in unirradiated cells/organisms after they were put in contact with the irradiated cells/organisms or immersed in the medium having previously been conditioned by the irradiated cells/organisms [9,10,11,12,13,14,15,16,17,18,19,20,21,22,23,24,25,26,27,28,29,30,31]. Adaptive response (AR) referred to mitigation of the biological effectiveness of a large challenging dose in cells/organisms through application of a small preceding priming dose [32], and the first in vitro study demonstrating AR was reported by Olivieri et al. [33]. Radiation-induced rescue effect (RIRE) on targeted irradiated cells/organisms referred to the benefits they derive from feedback signals released from non-targeted bystander cells/organisms [34,35,36]. Radiation-induced genomic instability (RIGI) referred to delayed lethal mutations or reproductive cell death, as well as elevations in the rate of de novo appearance of chromosomal aberrations and gene mutations, in the progeny of irradiated cells [37,38,39,40,41]. RIGI could be observed in progeny which were many generations after the initial irradiated cells, while the irradiated cells themselves or their immediate progeny could appear healthy but were in fact unstable and mutation-prone [13,42,43,44,45,46]. Changes in gene profiles induced by IRs were widely characterized through microarray hybridization techniques [47,48] with enhanced detection accuracy of differential expression [49]. These non-traditional effects caused by IRs had raised concerns about the low-dose radiation risk and the linearity of the relationship between cancer risk and radiation dose.

Over the past ten years, accumulating evidence has supported that zebrafish is an excellent in vivo model for studying the biological effects of IRs. Some of the findings on targeted effects as well as non-targeted effects are summarized in the following.

As regards IR-induced targeted effects, the study of Geiger et al. was among the first to ascertain the dose responses of zebrafish to γ-rays, which reported that the damage level and caspase activation in zebrafish were proportional to the radiation dose [50]. The dose responses of zebrafish embryos to low-dose α particles, protons, X-rays and neutrons were further established using apoptotic events in whole zebrafish embryos at 24/25 h post-fertilization (hpf) [51,52,53,54]. In particular, biphasic/triphasic dose responses were suggested as a common phenomenon in the low-dose regime [51,52,53,54]. A dose-response curve with a hormetic zone typically has a “J” shape or inverted “U” shape, and is thus nonlinear and biphasic, while a dose-response curve with an extra “subhormetic” zone in addition to a hormetic zone is “triphasic”. Gagnaire et al. studied the effects of γ-irradiation in zebrafish larvae by examining physical parameters such as mortality and hatching rate as well as several biomarkers such as reactive oxygen species (ROS) production and detoxification enzyme [55]. Their results showed that IR-induced oxidative stress led to DNA damages [55]. Ryan et al. identified a hyper-radiosensitive (HRS) response in the ZEB-2J (zebrafish cell line) cells upon γ-irradiation with a dose <0.5 Gy [56]. Guo et al. revealed that the p53 protein in mutant p53 zebrafish embryos upon ionizing radiation exposure maintained a very high level when compared to wild-type embryos, thereby suggesting that the p53 protein played a crucial role in IR-induced carcinogenesis [57]. Bladen et al. suggested that Ku70 and Ku80 were essential in repairing IR-induced DNA damages in zebrafish embryos through the nonhomologous end-joining (NHEJ) pathway [58,59]. Jaafar et al. studied the long-term effects of γ-rays in adult zebrafish and in zebrafish embryos in terms of gene expression in the liver tissue using Affymetrix microarray [60], and found that more than 300 transcripts were modified in the liver of adult zebrafish liver upon γ-irradiation with doses of 0.1 and 1.0 Gy [60]. More recently, Kong et al. showed that exogenous nitric oxide (NO) protected zebrafish embryos from damages induced by X-rays in a dose-dependent manner [61].

As regards IR-induced non-targeted effects, some of the findings on AR, RIBE and RIRE are summarized in the following. AR was shown to be successfully induced by α particles and protons in zebrafish embryos [62,63,64]. Interestingly, no AR was observed to be induced by neutrons [65], which was attributed to the presence of neutron-induced hormesis as well as γ-ray hormesis [65]. RIBE was demonstrated in zebrafish [66], while RIBE and RIRE were demonstrated in zebrafish embryos exposed to α particles [67,68]. Smith et al. demonstrated that RIBE could transcend taxonomic group (zebrafish and medaka) and trophic level in fish (irradiated California blackworms and non-irradiated rainbow trout), and suggested that RIBE signals could be transmitted through an ecosystem [69]. Saroya et al. [70] demonstrated that zebrafish injected with reserpine, an inhibitor of serotonin, before X-ray exposures could eliminate the RIBE. This suggested that serotonin was involved in the bystander signaling. RIBE arising from neutron irradiation was more equivocal. Ng et al. reported RIBE in naive zebrafish embryos partnered with zebrafish embryos having been irradiated with neutron doses between 20 and 50 mGy [71], while Wang et al. did not detect RIBE in naive zebrafish partnered with zebrafish having been irradiated with a neutron dose of ~100 mGy [72]. The different observations were attributed to different γ-ray hormesis arising from γ-ray contamination of the neutron sources [71]. RIBE was also shown to be totally suppressed by the NO scavenger 2-(4-carboxyphenyl)-4,4,5,5-tetramethylimidazoline-1-oxyl-3-oxide (cPTIO) [73], which suggested that NO was involved in the bystander response between zebrafish embryos. Interestingly, AR was also induced in bystander zebrafish embryos upon partnering with irradiated zebrafish embryos [74,75]. RIRE was observed in zebrafish embryos induced by α particles [68]. In relation, exogenous carbon monoxide (CO) was shown to be able to suppress RIBE [76], which agreed with previous results [77], but unable to suppress RIRE induced by α particles [76]. A previous study on cells revealed that CO could protect bystander cells from the toxicity of NO [78]. It was suggested that the bystander zebrafish embryos did not need NO-induced damages to initiate their release of the RIRE signals [76].

## 3. Epigenetic Effects of Ionizing Radiation

The relationship between epigenetics and IR was comprehensively reviewed [79,80,81]. Accumulating evidence has demonstrated that IR exposures can lead to epigenetic alterations, which play an important role in genomic instability that can affect the next generations [82,83,84]. Previous studies had confirmed DNA damages and chromatin alterations in the progeny of irradiated male mice [82,85]. On the other hand, epigenetic regulation can also be affected by mitochondrial functions, which are important in maintaining metabolic homeostasis and play a critical role in IR responses [86,87]. Recently, the impact of metabolic defects on epigenetic processes was summarized and the link between the mitochondrial function and epigenetics was outlined [88,89]. Shaughnessy et al. demonstrated that dysfunction of mitochondria affected epigenetic regulation [90]. The roles of mitochondria in cellular response to IR and in carcinogenesis-related epigenetics were comprehensively reviewed [91,92,93]. Accumulating evidence has indicated that ROS play a crucial role in epigenetic processes through the oxidative stress [94]. A cross-interaction between expression of miRNA and mitochondrial function was found, which suggested that epigenetic regulatory mechanisms were involved in RIGI arising from mitochondrial dysfunction [95]. Furthermore, Szumiel reviewed that mitochondrial (mt) DNA could be damaged by high levels of ROS generated by IRs [96]. The mutated mtDNA would affect the activity of methyltransferases and then lead to global DNA hypomethylation [96]. Collectively, these findings suggested that dysfunction of mitochondria would lead to RIGI by altering the DNA methylation status.

The first epigenetic alterations identified in cancer initiation and progression were changes in DNA methylation [97,98]. Epigenetic alterations and failures to properly maintain heritable epigenetic marks were suggested as potential causes of cancers [99,100]. In particular, in addition to genetic alterations, global epigenetic abnormalities were revealed in human cancer cells [99,101]. DNA methylation, histone modifications, and microRNA profiles that change during the initiation and progression of cancer were reviewed [102,103,104].

### 3.1. DNA Methylation

DNA methylation refers to the process in which a methyl group (CH_3_–) is added to the DNA, which often modifies the functions of the involved genes [105]. It is a major epigenetic modification in the genomes of higher eukaryotes. DNA methylation was the first identified epigenetic alteration and has been the most studied. The most extensively characterized DNA methylation process is the addition of the methyl group to the 5-carbon of the cytosine ring leading to the formation of 5-methylcytosine (5-mC), which can inhibit transcription [106,107]. In somatic cells, most 5-mC occurs at CpG sites within the DNA sequence (where a cytosine nucleotide and a guanine nucleotide are separated by only one phosphate, i.e., 5′-C-phosphate-G-3′) in form of a paired symmetrical methylation. However, in embryonic stem (ES) cells, a large amount of 5-mC is also found at non-CpG sites [108]. In the genomic DNA, most CpG sites are heavily methylated, except those in CpG islands (DNA sequences containing an atypically high frequency of CpG sites) in germ-line tissues and those close to promoters of normal somatic cells, which remain unmethylated to enable gene expression [109,110,111].

In general, there are three types of DNA methylation alterations, namely, hypermethylation, hypomethylation and loss of imprinting. DNA hypermethylation describes the situation when methylation occurs at sites that are unmethylated under normal circumstances. In carcinogenesis, hypermethylation of genes are involved in the cell cycle, DNA repair, angiogenesis, metabolism of carcinogens, apoptosis, and cell-cell interactions [112]. DNA hypomethylation usually refers to losses of DNA methylation in the genome, although it can also occur locally [113,114]. In relation, global DNA hypomethylation refers to the decrease in the total 5-mC content in the genome. DNA hypomethylation can participate in activation of proto-oncogenes [112,115]. Genomic imprinting refers to the epigenetic modification in which certain genes are expressed in a parental-origin-specific manner, while loss of imprinting describes the disruption of such an epigenetic modification. Loss of imprinting can result in gaining or losing of methylation or other chromosomal marks, or losing the pattern of parental-origin-specific gene expression [116].

DNA methylation is mediated by a family of enzymes called DNA methyltransferases (DNMTs). DNMTs are responsible for the establishment and maintenance of methylation patterns. In mammals, DNA methylation to form 5-mC is established by “de novo” DNA methyltransferases (DNMT3a, DNMT3b, and DNMT3L) and subsequently maintained by DNMT1 [117,118,119]. DNMT1 acted as the “maintenance” methyltransferase since it was the primary enzyme responsible for copying methylation patterns after DNA replication [118]. In addition, the DNMT3 family was classified as de novo methyltransferases since the involved methylation occurred at previously unmethylated cytosines [120].

Evidence has shown that direct IR exposure would affect DNA methylation patterns. For example, acute exposures to IR with low linear-energy-transfer (LET) values, such as X-rays and γ-rays, led to global DNA hypomethylation [121,122], which could occur at different genomic sequences including repetitive elements, retrotransposons, CpG-poor promoters, introns and gene deserts [123]. Global hypomethylation can contribute to carcinogenesis through favoring mitotic recombination which leads to deletions, translocations and chromosomal rearrangements, thereby causing genomic instability and fragility [99,124]. Kovalchuk et al. [125], Raiche et al. [126] and Pogribny et al. [127] showed that global hypomethylation upon an IR exposure would cause change in mice, depending on parameters such as sex, tissue and dose rate. This loss of methylation was also associated with radiation-induced alterations in the de novo methyltransferases DNMT3a and DNMT3b [126,127]. Interestingly, Bernal et al. revealed an increase in the DNA methylation level by generating ROS in the viable yellow agouti (A^vy^) locus using A^vy^ mouse model in the low-dose-radiation hormetic regime [128], where low-dose simulation is shown [129,130]. This hinted that radiation hormesis could be related to hypermethylation upon low-dose IR exposures [128]. As regards non-targeted effects, Rugo et al. [131] studied the effects of the irradiated cell conditioned medium (ICCM) on unirradiated cells. Interestingly, through comet analysis, inactive DNMT1 in mice ES cell could protect neighboring cells from indirect induction of genomic instability [131]. Furthermore, it was found that only those irradiated cells having normal DNMT1 and DNMT3a expression could induce bystander effects in naive bystander cells [131]. Koturbash et al. [132,133] found significant changes in the levels of DNA methyltransferases in bystander tissues in mice in vivo upon IR exposures and therefore proposed that epigenetic transcriptional regulation was involved in RIBE.

IR-induced global loss of DNA methylation was also found related to changes in histone methylation patterns (see also discussion on histone modifications below), specifically with the loss of histone H4 lysine tri-methylation [134]. Moreover, Koturbash et al. attributed the initiation and maintenance of downregulated DNMT3a in the bystander liver tissue in a mouse’s body to the significant upregulation of the microRNA *miR-194* [135] (see also discussion on microRNA profiles below).

### 3.2. Histone Modifications

There are four core histones, namely, H2A, H2B, H3, and H4, which modulate the normal epigenome to maintain gene expression patterns and normal chromosome structure and function [136]. The N-terminal domains of these four histones (terminated by a free amine group (–NH_2_)) and the C-terminal domains of H2A and H2B (terminated by a free carboxyl group (–COOH)) are poorly structured protrusions from the nucleosome called the “histone tails.” Histone tails provide sites for covalent modifications such as acetylation, methylation and phosphorylation. The structures and functions of the chromatin are determined by the combinations of various histone modifications and other chromatin-binding proteins. For example, histone tail modifications regulate the chromatin structure and gene expression [137]. Transcriptions are controlled by interactions between histone modifications and DNA methylation. IR-induced global loss of DNA methylation could be related to altered histone methylation patterns, in particular the loss of histone H4 lysine tri-methylation [134].

The major histone modifications are acetylation, methylation, phosphorylation, and ubiquitination [136]. Histone acetylation involves the transferal of an acetyl functional group (CH_3_CO–) to the lysine of a histone tail, and was reviewed in refs. [137,138]. Acetylation of lysines was found to be highly dynamic and regulated by opposing actions of two families of enzymes, namely, histone deacetylases (HDACs) and histone acetyltransferases (HATs). HDACs remove the acetyl group from acetyllysine (Ac-Lys) and then release an acetate molecule [139] so they are responsible for histone deacetylation, which is commonly associated with gene silencing. HATs transfer an acetyl group from the acetyl coenzyme A (acetyl-CoA) to the lysine residue [139] so they neutralize the positively charged lysines on the histone tails and weaken the interaction between histones and DNA [137]. On the other hand, histone methylation refers to the process in which a methyl group (CH_3_–) is introduced to the lysine or arginine of a histone tail, and mainly occurs on the side chains of lysines and arginines [137]. Histone methylation does not change the charge of the histone protein [137], but recruit silencing or regulatory proteins. Histone phosphorylation refers to the process in which a phosphate group (PO_4_–) is introduced to the serine or threonine of a histone tail. Most histone phosphorylation sites lie within the N-terminal histone tails and the levels of the modification are controlled by kinases and phosphatases [140]. Histone ubiquitination refers to the process in which an ubiquitin protein is introduced to the histone, which is mainly on the lysine residues that are located at the C-terminus of the H2B histone [141].

One of the most studied histone modifications, particularly those related to IR exposure, is the phosphorylation of histone H2AX at serine139 (γ-H2AX) [142]. Formation of γ-H2AX is one of the earliest cellular responses to DSBs induced by IR exposures [143,144], which is crucial for the repair of DSBs and for the maintenance of genome stability [142,143,144,145,146]. Chromatin immunoprecipitation (ChIP) is a powerful tool for characterizing covalent histone modifications and DNA–histone interactions in vivo [147,148,149] and is thus widely used to identify the presence of modified histones [150,151]. Protocols for ChIP assays have been well established for mammalian cells [152], yeast [153] as well as *Drosophila* [154].

### 3.3. MicroRNA Profiles

A family of non-coding RNA genes has been discovered in plants and animals, which are single-strand RNA molecules with lengths of about 18–22 nucleotides and are called microRNAs (or miRNAs) [155]. They act in a nuclear protein complex known as RNA-induced silencing complex (RISC). The miRNAs regulate gene expression post-transcriptionally and are expressed in a development and cell-type-specific manner [156]. They play a crucial role in various biological processes including development, proliferation, differentiation to cancer and apoptosis [157,158,159].

Metheetrairut and Slack [160] and Chaudhry [161] reviewed the involvement of miRNA in the response to targeted IR exposures, which included cell cycle arrest, proliferation and apoptosis. The IR-responsive miRNA profiles could be revealed by a variety of methodologies, including the real-time quantitative PCR [162,163,164], microarray [165,166,167,168,169,170,171,172,173,174,175,176] and deep sequencing techniques [177,178]. MicroRNA can also be involved in a non-targeted effect induced by IR such as RIBE. Koturbash et al. showed that partial irradiation of a rodent significantly upregulated *miR-194* in the bystander liver tissue, which explained the initiation and maintenance of the downregulation of DNMT3a and MeCP2 (methyl CpG binding protein 2) in the same bystander tissue [135]. MeCP2 is a protein in the MBD (methyl CpG-binding domain) family, and plays a critical role in methylation-mediated chromatin remodeling and gene silencing [3,179,180,181,182,183]. Moreover, when a three-dimensional artificial tissue was exposed to a microbeam of α particles, the proliferative state would be changed in bystander tissues, which was affected by the deregulation of miRNA expression [184].

## 4. Epigenetic Effects of Ionizing Radiation in Zebrafish

### 4.1. Zebrafish as an In Vivo Model

Zebrafish has been regarded as a suitable model organism in many biomedical studies, including developmental biology, cancer research and epigenetics [185,186]. Zebrafish is also widely considered as an excellent animal model for studying the molecular mechanisms underlying human diseases as it has vascular, hematopoietic, immune and central nervous systems, as well as organs with some phenotypic features resembling those features in humans [82]. In relation, the zebrafish embryo model has also become popular in studies on toxicology [187], developmental biology [185] and carcinogenesis [188].

### 4.2. Epigenetic Effects in Zebrafish

The epigenetic landscape in zebrafish embryos had been widely studied by microarray analysis [189,190,191]. Zebrafish embryos first develop from messenger RNAs (mRNAs) and proteins stored in the egg without ongoing transcription [192,193]. The epigenetic status changed rapidly during embryogenesis [194,195,196,197,198,199], which could efficiently control gene expression [200]. These features provide unique opportunities for studying epigenetic mechanisms. During the zygotic stage (from 0 to 0.25 h post-fertilization (hpf)), no apparent transcription was observed [200]. Within this development stage, many maternal mRNAs encoded for increasing histone modification. Recent studies showed that zebrafish embryos started establishing epigenetic mechanisms at the mid-blastula transition (MBT) during the 10th cell cycle (~3 hpf, 1000 cells), which was referred to as the zygotic gene activation (ZGA) [196,197]. At the MBT stage, the majority of maternal mRNAs were degraded with the aid of zygotic miRNAs (miR-430) [201]. At the gastrula stage, when the germline was formed (5.3 hpf, 50% epiboly), the genes started to be expressed [200,202]. The epigenetic status became more complex afterwards [193] as there was an increase in histone modification in genes [191,203] and the histone marks increased and decreased dynamically [203].

### 4.3. DNA Methylation in Zebrafish

DNA methylation mechanisms in fish are in general conserved with those of mammals [204,205,206,207]. Most of the effector proteins in the methylation machinery were identified in zebrafish [208]. Fang et al. found that global DNA methylation began to increase in zebrafish embryos after 3.3 hpf and reached a plateau between 6 and 96 hpf through measuring the global DNA methylation in zebrafish embryos using the MethylFlash™ Methylated DNA Kit (Epigentek Group, Farmingdale, NY, USA) [209]. Recently, whole zebrafish genome sequencing was conducted [6]. It was found that most zebrafish genes had mammalian orthologs, and most epigenetic regulators were highly conserved: there were 75% and 92% identity between zebrafish and human DNMT1 and HDAC1, respectively [186]. Nevertheless, three human DNMTs, i.e., DNMT3a, DNMT3b and DNMT3L, were involved in de novo DNA methylation, whereas six corresponding genes were involved in zebrafish [210,211]. Although the homologous genes of the DNMT3 family were identified in zebrafish, further experimentation would be required to reveal which were orthologs and which were paralogs. Besides, DNA hydroxymethylation is another epigenetic mark that modifies the cytosine at the fifth position by adding a hydroxymethyl group to DNA, and 5-hydroxymethylcytosine (5hmC) has been found to play a key role in the activation of DNA demethylation pathway [212]. A number of studies also reported that decrease in 5hmC led to cancer development [213,214,215]. Recently, the high-performance liquid chromatography mass spectrometry (LC/MS) method was employed to quantify the level of 5hmC, which showed no hmC-mediated reprogramming in zebrafish before 12 hpf [216]. This result agreed with previous findings through immunostaining, which reported non-detection of 5hmC during early zebrafish development [198,217].

Knocking-down of DNMT1 or DNMT3b by morpholino oligonucleotides in zebrafish embryos led to differentiation defects [218,219,220], which clearly demonstrated the importance of zebrafish DNMT enzymes for development. Besides, Mortusewicz et al. showed that DNMT1 was required not only for DNA synthesis, but also for DNA repair [221]. In addition, Ha et al. used near-infrared laser micro-irradiation to demonstrate that DNMT1 modulated the repair rate of double strand breaks (DSBs) and was essential for suppressing abnormal activation of DNA damage response in the absence of exogenous damages [222]. The results suggested that DNMT1 acted as a regulator of genome integrity and as an early responder to DNA DSBs [222].

Recently, Pereira et al. showed that γ irradiation increased the global methylation in both irradiated and bystander embryonic zebrafish fibroblast (ZF4) cells through mass spectrometry analyses [223]. High performance liquid chromatography–tandem mass spectrometry (HPLC–MS/MS) has been a common technique to evaluate the relative degree of global DNA methylation through determining the genome-wide level of cytosine and 5-mC in DNA samples [224]. On the other hand, Geiger et al. demonstrated that temozolomide, a DNA methylating agent, enhanced the radiosensitivity of U251 human glioma cells when transplanted into zebrafish embryos, while temozolomide treatment did not lead to discernible effects on development of the zebrafish embryos [225]. Taken together, previous evidence suggested that modifications in DNA methylation occurred in zebrafish when they were exposed to IR.

Genome-wide DNA methylation levels in zebrafish can be quantified through methylated DNA immunoprecipitation (MeDIP) and shotgun bisulphite sequencing. The protocols and analytical methods applicable in zebrafish studies were reviewed by Wu et al. [226]. The MeDIP technique was first described by Weber et al. [227] to reveal regional methylation. The principle was to subject the methylated DNA to a “tiling” microarray containing oligonucleotide probes that tiled the whole genome or all promoters, which was called MeDIP-chip [228]. On the other hand, bisulphate sequencing [229,230] revealed DNA methylation at base-pair resolution. Bisulphite treatment of genomic DNA coupled with DNA sequencing has been regarded as a standard approach to detect individual methylated cytosine residues.

### 4.4. Histone Modifications in Zebrafish

During the first cell cycles of the zebrafish embryos, some histone marks such as H3K4 (H3: H3 family of histones; K: standard abbreviation for lysine; 4: position of amino acid residue counting from the N-terminus), H3K9 or H3K27 were acquired for epigenetic reprogramming [203,231]. Cayuso et al. reviewed the roles of chromatin modifications in zebrafish development and regeneration [232]. At the MBT stage, genomic confirmation was mainly acquired by histone modifications [191,196,203,233]. From the MBT stage onwards, there were increases in H3K4me3 levels (me3: 3 methyl groups added) and H3K27me3 levels [191,196,233]. H3K4me3 mainly marked housekeeping genes while H3K27me3 occupied developmentally regulated genes [191]. In a separate study, loss of HDAC1 was found to increase global histone acetylation in zebrafish development [234]. In relation, Lindeman et al. reported a protocol for immunoprecipitation of modified histones from mid-term blastula zebrafish embryos [235]. Chromatin immunoprecipitation (ChIP) is a widely used method for identifying the presence of modified histones through quantitative PCR (ChIP-qPCR) or hybridization of ChIP DNA to microarrays (ChIP-chip) at as early as the 256-cell stage [203]. This tool would help studies on histone modifications by IR in zebrafish.

### 4.5. MicroRNA Profiles in Zebrafish

Inactivation of miRNA biogenesis was found to cause severe defects in zebrafish embryos [236]. Chen et al. recorded the miRNA profiles during the development of zebrafish [237] and examined 154 different miRNAs in zebrafish at different stages from the early zygote period (0 hpf) to several months old. The authors used the total RNA isolated from zebrafish at different developmental stages to clone and sequence small RNA libraries [237]. The information revealed that miRNAs were absent in the early zygote stage, which was commensurate with the findings for the African clawed frog, *Xenopus laevis*, by Watanabe and colleagues [238]. Watanabe et al. examined a total of 24 known miRNAs of *Xenopus laevis* during development from the oocyte to tadpole stage, and showed that most (17 of 24) miRNAs emerged at a specific stage and were continuously expressed until the tadpole stage, so the authors suggested that these miRNAs were involved in differentiation [238]. In relation, Chen et al. found that in zebrafish embryos, a zebrafish-specific miR-430 family started to express at 4 hpf and this family’s clusters dominated the miRNA profile up to 24 hpf [237]. The miR-430 family was related to human and mouse ES cell-specific miRNAs [239,240]. On the other hand, miR-125b, one of the miRNAs, was shown to directly regulate p53 in zebrafish and in human cells [241]. A significant down-regulation of miR-125b expression was found in zebrafish embryos in response to IR-induced DNA damages, which resulted in a rapid increase in p53 protein [241]. Accordingly, Le et al. suggested that miR-125b-mediated regulation of p53 was critical for modulating apoptosis in human cells as well as in zebrafish embryos exposed to IRs [241].

MicroRNAs could be obtained by locked-nucleic acid (LNA)-modified DNA oligonucleotide probes [242]. LNAs refer to a class of RNA analogs containing a 2′-*O*,4′-*C*-methylene bridge in such a way that the ribose rings are “locked” to enhance sensitivity and specificity. LNA probes are very specific to mature miRNAs [243,244,245,246,247] and Wienholds et al. used LNA probes for in situ whole-mount hybridization detection of conserved vertebrate miRNAs in zebrafish embryos [242]. Recently, analyses of miRNAs with higher efficiency and lower cost were also achieved by in situ hybridization (ISH) using conventional digoxigenin-labeled riboprobes in both whole mounts and histological sections in zebrafish embryos [248]. In addition, using riboprobes directed against the primary miRNA transcript could distinguish among expression patterns from different miRNA genes which encoded the same mature miRNA [248].

## 5. Conclusions

Accumulating evidence has shown that IR-induced damages lead to transgenerational genomic instabilities [249,250,251,252]. Such genomic instabilities were deemed to associate with ionizing radiation (IR)-induced epigenetic alterations. The present paper reviewed the potential of the zebrafish, *Danio rerio*, as an in vivo model to assess the epigenetic effects of IR. Embryogenesis is a stage which has rapid changes in the epigenetic status and which is markedly radiosensitive, so zebrafish embryos are in particular ideal for studying epigenetic mechanisms and for evaluating responses to IR [30,253].

In the present review, the epigenetic effects of IR studied or potentially assessed using the zebrafish model included DNA methylation, histone modifications and miRNA expression, which had already been identified in zebrafish [194,195,196,197,198,199,203,209,210,211,237]. A summary of tools for analyzing epigenetic changes in zebrafish is given in Table 1.

As regards IR-induced DNA methylation changes, γ irradiation increased the global methylation in irradiated and bystander embryonic zebrafish fibroblast ZF4 cells [223], while the radiosensitivity of U251 human glioma cells transplanted into zebrafish embryos was enhanced by the DNA methylating agent temozolomide [225]. Considering other in vivo models, IR exposure induced global hypomethylation in mice, which depended on parameters such as sex, tissue and dose rate, and was associated with alterations in DNMT3a and DNMT3b [125,126,127]. IR exposure also led to significant changes in the levels of DNA methyltransferases in bystander tissues in mice [132,133].

As regards IR-induced miRNA expression, a significant down-regulation of miR-125b expression was found in zebrafish embryos in response to IR-induced DNA damages, which led to a rapid increase in p53 protein [241]. Considering other in vivo models, IR was found to induce significant changes in the levels the microRNA *miR-194* in bystander tissues in mice [135] and to deregulate miRNA expression in three-dimensional bystander artificial tissues [184].

Interestingly, despite the availability of tools for analyzing histone modification changes in zebrafish and in other in vivo models, no in vivo studies on IR-induced histone modification changes have been reported to date. As explained in the Introduction, studies on IR-induced epigenetic effects using zebrafish have just begun, and as such results have not yet been plentiful. Considering the ubiquitous presence of IR in our environment and the accumulating evidence showing that IR exposures induce epigenetic alterations, and given that zebrafish has become a popular vertebrate model for studying molecular mechanisms underlying human diseases, it is expected the zebrafish model can be extensively employed as an in vivo model to assess epigenetic effects of ionizing radiation in the near future.

## Figures and Tables

**Table 1:** A summary of tools for analyzing epigenetic changes in zebrafish, including those for detecting genome-wide DNA methylation changes, histone modification changes as well as miRNA expression.

Tool	Mechanisms/Characteristics	References
A. For detecting genome-wide DNA methylation changes in zebrafish		
High performance liquid chromatography–tandem mass spectrometry (HPLC–MS/MS)	Quantify global levels of 5-methylcytosine (5-mC) and 5-hydroxymethylcytosine (5-hmC) in DNA sample.	[223]
	Very high sensitivity.	
Bisulphite sequencing	Reveal DNA methylation at base-pair resolution.	[226]
	Global measurement of DNA methylation.	
	Can be coupled with high-throughput sequencing.	
Methylated DNA immunoprecipitation (MeDIP)	Enrich for methylated DNA sequences.	[226]
	Can be coupled with either high-resolution array hybridization or high-throughput sequencing.	
Methylated DNA Quantification Kit	Quantify 5-methylcytosine (5-mC) content or global methylation.	[209]
	Easy to use.	
B. For detecting histone modifications in zebrafish		
Chromatin immunoprecipitation (ChIP)	Well-known established protocol in zebrafish embryos.	[203,235]
	Can be coupled with quantitative PCR (ChIP-qPCR) or hybridization of ChIP DNA to microarrays (ChIP-chip).	
C. For detecting microRNA in zebrafish		
Locked-nucleic acid (LNA)-modified DNA oligonucleotide probes	Target specificity and sensitivity.	[242]
	Good for detection of short RNA, e.g., miRNAs.	
Conventional digoxigenin-labeled riboprobes	Can be used for whole mounts and histological sections in zebrafish embryos.	[248]
	Higher efficiency and lower cost than LNA probes.	
